# If Freud had known…

**DOI:** 10.3389/fpsyg.2025.1556279

**Published:** 2025-05-20

**Authors:** Marion Hendrickx, Quentin Tousart, Emmanuel Drouin

**Affiliations:** ^1^Psychiatry Service, Lille Catholic Institute Hospital Group (Groupe Hospitalier de l'Institut Catholique de Lille), GHICL, Hôpital Saint Vincent de Paul, Lille, France; ^2^Société Webpulser, Mons-en-Baroeul, France; ^3^Neurology Service, Lille Catholic Institute Hospital Group (Groupe Hospitalier de l'Institut Catholique de Lille), GHICL, Hôpital Saint Philibert, Lomme Cedex, Lille, France

**Keywords:** Freud, Einstein, hysteria, conscious, unconscious, Einstein's theory, AdS/CFT duality, quantic

## 1 Introduction

Neurology and psychiatry maintain a complicated relationship (Drouin et al., 2022), which continues to question the fundamental interaction between mind and matter. How does the brain manufacture the mind? The nature of consciousness has been an enigma for millennia, challenging philosophers, physicians, and scientists alike. New research suggests that the brain functions as a confederation of independent modules working together in a layered architecture. Understanding how consciousness emerges from such an organization requires reverting to the fundamentals of physics. It also necessitates examining the transition from inanimate matter to living systems if we are to bridge the divide between brain and mind (Gazzaniga, 2022). As John Searle aptly noted, studying the brain without studying consciousness would be like studying the stomach without studying digestion (Searle, 2000).

At the center of this question stands the figure of Sigmund Freud (1856–1939). His career illustrates the transition from a conception of mental disorders as purely organic to a psychodynamic approach based on the hypothesis of an unconscious. Originally trained as a neurologist in the clinical anatomical tradition of the nineteenth century, Freud progressively developed a theory of the unconscious. This theory moved him away from the medical materialism of his time. Importantly, Freud was not a dualist who defended the idea that psychic life exists outside our brains. Rather, he sought different explanatory frameworks when the neuroscience of his era proved insufficient.

More than a century after the emergence of psychoanalysis, advances in neuroimaging and modern physics invite us to reconsider the perceived separation between the organic and the psychic. The connectome, defined as the comprehensive mapping of neural connections in the brain (Kruper et al., 2024), can now be visualized through diffusion MRI. This technology reveals structural modifications in various psychiatric pathologies. Simultaneously, contemporary physics such as quantum physics or relativity has opened new perspectives on the nature of consciousness. This has occurred particularly through concepts like the AdS/CFT duality (Anti-de Sitter/Conformal Field Theory), which establishes a mathematical correspondence between gravitational theories and quantum theories (Awret, 2022).

These developments prompt us to ask: should Freudian thought be revisited in light of these new discoveries? Our aim is not to “rehabilitate” Freud but rather to examine how the physical and neuroscientific frameworks of our time might provide new interpretations of his key insights on the functioning of the psyche.

This article offers an examination of this question by first briefly retracing Freud's shift from neurology to psychoanalysis. Second, we analyze how modern neuroscience illuminates certain Freudian concepts while revealing their neurobiological underpinnings. Third, we critically examine aspects of Freudian theory requiring substantial revision. Fourth, we explore how concepts from physics could enlighten our understanding of consciousness and some Freudian concepts, before proposing an integrative approach to these complex phenomena.

We will see how the traditional separation between mental processes and physical brain function—commonly referred to as the mind-body dichotomy—tends to become less relevant in light of contemporary scientific frameworks.

This perspective would perhaps not have surprised Freud himself. Despite his apparent distancing from neurology, Freud never completely excluded the possibility of a future biological understanding of the phenomena he described: “*Given the intimate dependence that exists between the things that we divide into corporeal and psychic, we can foresee that a day will come when the paths will open to knowledge and also, hopefully, to practice, leading from biology organs and chemistry in the field of manifestations of neuroses*” (Freud, 1926, translation by the authors). What he could not imagine was that physics itself would also provide avenues for rethinking the relationships between matter and consciousness.

## 2 Freud: from neurology to psychoanalysis

Freud's intellectual journey from neurology to psychoanalysis represents a significant epistemological shift in the history of medicine and psychology. In October 1885, Freud, then aggregated to neurology, arrived in Paris. He spent several months working in the service of Professor Jean-Martin Charcot (1825–1893) at La Salpêtrière. Freud was particularly drawn to Charcot's unconventional approach of using hypnosis to study hysteria. This condition was largely dismissed by the Viennese medical community, which adhered strictly to organic explanations of mental disorders (Drouin et al., 2023).

The transition in Freud's thinking is particularly evident in his 1893 article published in the Archives of Neurology. In this work, he compares organic and hysterical paralysis. Initially approaching the topic from a neurological perspective, Freud progressively introduces the need for a psychological explanation: “Hysteria acts in its paralysis and other manifestations as if anatomy did not exist, or as if it did not have any consciousness” (Freud, 1893). This statement marks a crucial turning point in his thinking. He then asks permission to “enter the field of psychology” to explain how “there can be functional alteration without concurrent organic lesion” (Freud, 1893).

In suggesting that hysteria behaves as if anatomy does not exist, Freud established a theoretical foundation for exploring psychological processes independently of their neurological substrates. However, this should not be misinterpreted as a complete rejection of the biological basis of mental phenomena.

This departure from strictly neurological explanations represented a break with the medical thought of his time. It paved the way for a new conception of mental disorders where the psyche was not merely subordinate to anatomy but constituted a full field of investigation in its own right. Nevertheless, Freud maintained that the apparent independence of psychological processes from their neurological basis was a matter of current scientific limitations rather than an inherent dualism. As he would state in 1939: “the psychic topics of psychoanalytic theory have nothing to do with the anatomy of the brain” (Naccache, 2022). This statement reflected the methodological separation he established rather than an ontological claim about the independence of mind from brain.

Freud's shift from neurology to psychoanalysis thus established a conceptual foundation that would allow for the exploration of psychological phenomena on their own terms. This foundation now serves as a productive starting point for examining how contemporary neuroscience might bridge the gap between psychological and neurobiological explanations of mental phenomena.

## 3 Modern neuroscience and neuroscientific reinterpretations of Freudian concepts

### 3.1 The connectome: mapping neural connectivity

Recent advances in neuroscience and neuroimaging provide new perspectives on the functional/organic duality that Freud grappled with. The concept of the connectome—the comprehensive mapping of neural connections within the brain—has emerged as a crucial framework for understanding brain function and dysfunction (Kruper et al., 2024). Modern connectomics aims to establish the complete “wiring diagram” of the human brain using diffusion MRI (dMRI) to trace white matter fibers (tractography) and functional MRI to map connectivity patterns.

The emergence of diffusion MRI has been particularly revolutionary in revealing alterations to white matter organization in psychiatric pathologies (Le Bihan, 2003). Unlike molecular biology, which sometimes identifies the genetic origins of certain psychiatric disorders, neuroimaging targets structural anomalies that could underpin the phenotypic expression of numerous mental disorders.

Comparative multimodal meta-analyses have identified specific white matter abnormalities across various psychiatric conditions. For instance, both schizophrenia and bipolar disorder share anomalies in the corpus callosum, indicating a common deficit in connectivity, though these abnormalities are more severe in schizophrenia (Zhao et al., 2022). As we will see later, this is also the case for pathologies closer to Freud's interests, such as post-traumatic stress disorder or conversion disorders. These findings provide tangible evidence of structural differences underlying diverse psychiatric presentations, offering a neurobiological dimension to conditions that Freud approached from a psychological perspective.

### 3.2 Neural plasticity and therapeutic change

Perhaps most remarkable is the emerging evidence that psychotherapeutic interventions can lead to measurable changes in brain architecture.

A recent comprehensive meta-analysis by Cera et al. (2022) examining neural correlates of different psychotherapeutic approaches through fMRI provides strong empirical support for these observations. Analyzing 38 studies with a total sample of 1,688 subjects, they found that all forms of psychological intervention influenced brain function, with consistent changes in frontal, prefrontal regions, insular cortex, and superior and inferior frontal gyri. Importantly, their analysis revealed that psychodynamic approaches were distinctively associated with changes in the right superior and inferior frontal gyri and putamen, suggesting unique neurobiological mechanisms compared to CBT (Cognitive Behavioral Therapy) and mindfulness-based interventions. These findings align with the theoretical foundations of psychodynamic therapy, which emphasizes work with memory, space-time navigation, and representation—functions associated with these specific neural regions (Cera et al., 2022).

Similarly, electroconvulsive therapy for depression has been shown to modify white matter microstructure, with measurable improvements in neural fiber organization and connectivity following treatment. These changes were followed by relative “remyelination” under treatment, confirming the plasticity of white matter in response to therapeutic intervention, both psychotherapeutic and physical (Belge et al., 2022).

These discoveries do not reduce the psyche to its neuronal substrate. Rather, they demonstrate a dynamic interaction between psychological processes and brain structure. This relationship aligns unexpectedly with certain intuitions Freud had about the plastic and dynamic character of mental functioning (Freud, 1926). The convergence suggests that Freud's shift toward psychological explanations was not a rejection of biology but rather a recognition that a different level of analysis was necessary to capture the complexity of mental phenomena.

Modern neuroscience has begun to provide empirical validation for certain Freudian concepts. Three areas in particular demonstrate this convergence between psychoanalytic intuition and neuroscientific discovery: mechanisms of dissociation, memory suppression, and the neurobiological basis of conversion disorder.

### 3.3 Dissociation: from hypnosis to trauma-related disorders

Dissociation refers to a disruption in the usually integrated functions of consciousness, memory, identity, or perception of the environment. Operationally, it manifests along a spectrum from common experiences such as daydreaming to pathological conditions like dissociative identity disorder. Clinically, dissociation can be measured through standardized instruments that assess depersonalization (feeling detached from oneself), derealization (experiencing the world as unreal), psychoform dissociation (psychological disconnection), and somatoform dissociation (physical manifestations of psychological disconnection; De Pascalis, 2024). Freud initially conceptualized dissociation through his work with hypnosis and hysteria, viewing it as a psychological defense mechanism that segregates traumatic or conflicting mental content from conscious awareness (Appourchaux, 2014).

De Pascalis (2024) reviewed the neurofunctional correlates of hypnotic states, finding that hypnosis produces specific modifications in brain networks related to control and self-awareness. Imaging studies show decreased functional connectivity during hypnosis between frontal executive regions (executive control network) and other brain areas, corresponding to the reduction in agency and critical thinking during trance. EEG recordings also reveal particular oscillations (increased theta bands) associated with hypnotic susceptibility. These observations support dissociative theories of hypnosis, where certain voluntary control functions are “disconnected.” Thus, a phenomenon once viewed as “mysterious” (the loss of conscious control under hypnosis) finds a neurobiological explanation involving frontal decoupling that alters normal information integration in the brain.

This frontal decoupling mechanism appears to have parallels in trauma-related dissociative disorders. Dimitrova et al. (2025) examined white matter integrity in dissociative identity disorder (DID) and post-traumatic stress disorder (PTSD), finding reduced fractional anisotropy in bilateral pallidum, midbrain, and pontocerebellar white matter in DID patients compared to healthy controls. Fractional anisotropy is a measure derived from diffusion MRI that quantifies the directionality of water molecule movement in brain tissues. It serves as an indicator of the structural integrity of white matter tracts, reflecting the organization of nerve fibers and their myelination. Reduced fractional anisotropy reflects compromised white matter structural integrity, suggesting disrupted axonal organization or myelin sheath alterations that may impair efficient neural communication between brain regions. In both DID and PTSD groups, fractional anisotropy measurements in these white matter regions showed significant negative correlations with clinical measures of depersonalization, psychoform and somatoform dissociation, as well as trauma severity scores.

Thus, these studies have redefined dissociation operationally as a measurable alteration in neural connectivity patterns, particularly involving disruptions between frontal executive control networks and other brain regions. What Freud conceptualized as psychodynamic processes of dissociation now appears to involve specific patterns of altered connectivity between brain regions responsible for self-awareness, emotional regulation, and executive control.

### 3.4 Memory suppression and repression

Before exploring memory suppression mechanisms, it is essential to clarify the conceptual framework within which unconscious processes are understood in contemporary interdisciplinary discourse. As Naccache clarifies (in Appourchaux, 2014), any discussion of unconscious processes requires distinguishing between two fundamentally different concepts often confused in interdisciplinary discourse. The cognitive unconscious studied by modern neuroscience encompasses all information processing that occurs without conscious awareness yet influences behavior—including subliminal perception, implicit memory, and automatic motor control. This contrasts sharply with the Freudian dynamic unconscious, which specifically refers to mentally represented content actively kept from consciousness due to its conflictual or emotionally disturbing nature. This distinction is methodologically important: cognitive unconscious processes can be objectively measured through neuroimaging and behavioral paradigms, while the dynamic unconscious has traditionally been accessed through clinical techniques like free association. The neural mechanisms of memory suppression that we will describe provide a potential bridge between these concepts, suggesting that what Freud identified through clinical observation might have identifiable neural correlates (Salvador et al., 2018).

Indeed, the Freudian concept of repression—the unconscious exclusion of painful memories or thoughts from consciousness—finds a neurobiological correlate in contemporary research on memory suppression. Using the “Think/No-Think” paradigm, an experimental procedure for the study of intentional forgetting of unwanted memories, researchers have identified the neural underpinnings of a two-phase memory suppression process in which participants actively inhibit the recall of traumatic images (Song et al., 2024).

Functional MRI studies reveal that when suppressing a painful memory, the dorsolateral prefrontal cortex strongly activates and inhibits the hippocampus, the seat of episodic memory. This top-down mechanism reduces the intrusion of the memory into consciousness, functionally resembling the Freudian concept of repression—the blocking of an unbearable representation. After suppression, researchers observe decreased reactivation of emotional circuits, suggesting a weakening of the memory's affective charge. These data provide a concrete neurobiological correlate to the classic psychoanalytic concept of dynamic unconscious resulting from inhibition of affectively charged memory traces (Song et al., 2024).

### 3.5 Neurobiological basis of conversion disorder

Freud's early work on hysteria (now termed conversion disorder or functional neurological disorder) is finding validation through contemporary neuroimaging. Feinstein and Voon (2021) analyzed conversion disorder using functional MRI and found that patients with functional motor symptoms show a distinctive pattern of “bottom-up” limbic hyperactivity, particularly in the amygdala and emotional regions, coupled with deficient top-down regulation by the prefrontal cortex.

This neurobiological pattern suggests that intense emotion is no longer properly moderated by executive centers, leading to physical symptoms (paralysis, non-epileptic seizures) without organic lesion. Researchers also observe abnormal connectivity between the amygdala and motor cortex, providing a mechanistic model: excessive affect “short-circuits” voluntary motor control. These findings confirm in modern terms Freud's intuition of a conversion of psychic distress into bodily symptoms via a neurological mechanism of aberrant inhibition/excitation.

### 3.6 From psychological observation to neural mechanism

These examples demonstrate how certain Freudian insights can be reinterpreted through contemporary neuroscience without invalidating their clinical utility. The neural mechanisms underlying dissociation, repression, and conversion provide a biological foundation for phenomena that Freud described through purely psychological terms. This suggests a continuity rather than opposition between his observations and modern neuroscience.

What emerges is a more integrated understanding where psychological processes are seen as emergent properties of neural activity, but not reducible to it. The structural and functional brain alterations observed in various conditions align with Freud's clinical observations while providing a level of mechanistic explanation that was unavailable in his era. Far from diminishing the value of his psychological insights, neuroimaging has often validated them by providing the biological counterpart to processes he inferred from clinical observation.

The ability of psychotherapeutic interventions to induce measurable changes in brain structure further bridges the gap between psychological and biological levels of explanation. These findings suggest that the therapeutic methods Freud pioneered may work in part by facilitating neuroplastic changes that reorganize dysfunctional neural circuits—a biological mechanism he could not have known but intuitively grasped in his conception of therapy as a process of psychological reorganization.

## 4 Critical revisions of Freudian theories

While certain Freudian concepts find validation in modern neuroscience as we have just seen, it is necessary to acknowledge that other fundamental aspects of the original psychoanalytic theory have required substantial revisions. These revisions have occurred both under the impetus of scientific advances and through the internal evolution of the psychoanalytic discipline itself.

The theory of universal psychosexual development (oral, anal, phallic stages) has been largely supplanted in contemporary psychoanalysis by object relations theories. These theories include those of Klein, Winnicott, and Fairbairn. They emphasize the importance of early relationships rather than erogenous zones as psychic organizers (Kandel, 1998, 1999; Brusset, 2007). A particularly significant turning point was Freud's own abandonment of his traumatic seduction theory. This theory attributed the origin of neuroses to actual sexual abuse. Freud abandoned it in favor of the theory of Oedipal fantasy. Contemporary psychoanalysis has reconsidered this reversal in light of knowledge about childhood trauma (Haesevoets, 2003).

The Freudian unconscious, initially conceptualized as a reservoir of repressed drives, has been reconceptualized in modern psychoanalytic approaches. It is now seen as more relational and intersubjective. This moves it closer to the multiple non-conscious processing systems identified by cognitive neuroscience (Appourchaux, 2014). The exclusively psychogenic etiology of mental disorders has been abandoned in favor of multifactorial models. These models integrate discoveries in genetics and neurobiology. Even within the psychoanalytic community, researchers now recognize the importance of biological factors in disorders such as schizophrenia (Hibar et al., 2018).

The cathartic technique of emotional discharge, central to early Freudian therapies, has given way to an approach centered on the therapeutic experience, transference, and psychic elaboration. This aligns with research showing that cognitive restructuring is essential to trauma treatment (Abi-Dargham et al., 2023). Finally, the rigidity of the Oedipus complex has been softened in contemporary formulations. These formulations take into account the diversity of family and cultural configurations. The concept now corresponds to the subject's acceptance of limits and structuring prohibitions. This enables access to otherness and the construction of a differentiated subjectivity (Laufer, 2021).

It is important to note that while our article focuses on how insights from physics and neuroscience might provide new interpretations of Freudian concepts, the critical revisions described above stem primarily from developments in other fields. These include developmental psychology, trauma research, cultural studies, and the evolution of psychoanalytic theory itself, rather than directly from neuroscience and physics. The dialogue between psychoanalysis and these diverse fields has been ongoing throughout the past century, independent of recent advances in physics, and has contributed significantly to the refinement of psychoanalytic theory and practice.

These criticisms and evolutions of Freudian concepts highlight the limitations of a purely psychological or even neurobiological approach to consciousness and mental phenomena. It is precisely in these boundary areas that contemporary physics can offer complementary theoretical frameworks capable of enriching our understanding of these phenomena, in particular modern theories of consciousness, which we will now address.

## 5 Consciousness: contemporary models

### 5.1 Physics and consciousness

Contemporary sciences offers profound new frameworks for understanding consciousness. These frameworks potentially bridge the gap between mental processes and physical matter that has long challenged both neuroscience and philosophy.

To clarify these complex but essential physical concepts, general relativity, developed by Einstein, describes gravity as a curvature of space-time caused by mass and energy. It explains how space, time, and gravity are interconnected, revealing that time slows down near massive objects or at high speeds. It shows that space and time are not absolute but form a unified fabric that can bend and stretch, with gravity arising from this curvature. Quantum mechanics, on the other hand, explains the behavior of particles on a microscopic scale according to probabilistic principles. It describes how the smallest particles in our universe behave, following rules very different from everyday objects. Unlike the predictable physics of our daily life, quantum particles exist in states of probability until measured, behaving as both particles and waves. These two theories, although extraordinarily precise in their respective fields, have long been considered incompatible. The AdS/CFT (Anti-de Sitter/Conformal Field Theory) duality proposed by Maldacena (1999) offers a mathematical bridge between these theories. It establishes a correspondence between a gravity-free quantum theory (CFT) operating on a “frontier” and a gravitational theory in higher-dimensional space (AdS). This holographic correspondence means that the two descriptions, although formulated differently and in different dimensions, represent the same underlying physical reality (Le Bihan, 2023).

The holographic principle proposes that the information contained within a volume of space can be entirely encoded on the surface that bounds this region. Just as a two-dimensional hologram can store and project a three-dimensional image, this principle suggests that a physical theory in n dimensions can be exactly equivalent to a different theory operating in n-1 dimensions (Awret, 2022).

Building upon these foundational concepts in physics, Denis Le Bihan's theory applies this holographic principle to consciousness, proposing a radical reconceptualization of how mind emerges from brain (Le Bihan, 2023). He demonstrates that, at the connectome scale, principles of both special and general relativity are at work. The AdS/CFT duality provides a solution to the separation between quantum mechanics and general relativity (gravity). It does this by considering how gravity in a 5-dimensional curved spacetime naturally emerges from quantum phenomena occurring on its 4-dimensional “edge” or boundary. This principle forms the foundation of the holographic principle in physics. According to this principle, gravity appears like a hologram projected from the 4D quantum world.

Applying this framework to consciousness, Le Bihan proposes that consciousness emerges as a 5-dimensional hologram from the 4-dimensional neuronal activity of the cerebral cortex. In this model, Consciousness = gravity = hologram in 5D spacetime arising from cortical activity in 4D spacetime without consciousness. This approach proposes that the spacetime of our cerebral connectome exists in five dimensions rather than four. The fifth dimension allows for the natural, immaterial emergence of consciousness as a dual form of the 4D spacetime embedded in our material cerebral cortex (Le Bihan, 2023).

As Le Bihan explains, consciousness emerges “naturally” from spacetime by adding an extra dimension. The general idea is that the nature of consciousness escapes our complete understanding because we are unable to “see” this extra dimension (Le Bihan, 2022, 2023). This perspective resonates with Suominen's much earlier vision (1950). Suominen emphasized that consciousness experienced as “a single personal self” is an inner phenomenon (mind-mind) inseparable from behavior, an outer phenomenon (mind-body). Suominen had intuited the importance of time and its relativity in explaining consciousness based on Einstein's theory. However, he applied the physical theory literally rather than considering its application to the “speed of the brain” rather than the speed of light (Suominen, 1950).

### 5.2 Other modern theories of consciousness

Modern theories of consciousness offer sophisticated frameworks that can enrich our understanding of Freudian concepts. The Global Neuronal Workspace (GNW) theory, developed by Dehaene and Changeux, provides a neurobiologically grounded model of consciousness. This theory proposes that consciousness emerges when specific information is amplified, selected, and widely broadcast through a network of long-range neurons connecting multiple brain regions, particularly in the frontal and parietal cortices This “global broadcasting” makes information simultaneously accessible to various specialized brain systems—including those responsible for attention, memory, language, and action planning—thereby enabling conscious processing. The model explains neuroanatomical and EEG evidence showing that consciously perceived stimuli induce sustained, synchronized fronto-parietal activation patterns that are notably absent during subliminal perception (Mashour et al., 2020). The GNW theory also accounts for the well-known attentional capacity limit of consciousness—only a limited amount of information can enter the workspace simultaneously.

In contrast, Tononi's Integrated Information Theory (IIT) starts from phenomenological axioms (consciousness is intrinsic, unified, informative, etc.). It deduces the minimum physical properties a system must have to realize them (Albantakis et al., 2023). IIT proposes that a system possesses consciousness to the extent that it generates more information as a unified whole than the sum of its parts. Unlike GWS, IIT focuses on the intrinsic quantity of organized information in a system. This is independent of access or report.

In our view, these theories can be placed in dialogue with psychoanalytic concepts. For instance, the GWS theory's notion of information broadcast and integration resonates with Freud's ideas about preconscious processes becoming conscious. Similarly, IIT's emphasis on integration aligns with Freud's understanding of the synthetic function of the ego. We'll now take a closer look at a few other links often put forward in the literature between Freudian psychoanalysis and contemporary physics.

## 6 Physics perspectives on Freudian concepts

While the previous sections introduced how modern physics conceptualizes consciousness generally, we now turn to how specific physical theories might illuminate particular Freudian concepts. This section explores how specific Freudian concepts find resonance in modern physical theories, particularly those related to quantum mechanics, relativity, and the more recent holographic principles. In this section, we will address the topographical model, qualities of dreams and the analytic relationship.

### 6.1 Topography revisited: from psychic provinces to extra dimensions

Freud's “topographic” model conceptualized the psyche as having distinct regions—conscious, preconscious, and unconscious—suggesting a spatial organization of mental content. This metaphorical geography can find unexpected resonance in modern physics, particularly through the lens of the AdS/CFT duality.

As we explained above, the AdS/CFT correspondence establishes a mathematical equivalence between a gravitational theory in a higher-dimensional curved space and a quantum field theory on its boundary. The information contained in the “bulk” (higher-dimensional) description is completely encoded on the lower-dimensional “boundary.” This holographic principle suggests that seemingly separate descriptions may represent the same underlying reality viewed from different perspectives or dimensional frameworks (Awret, 2022).

Awret (2022) and Le Bihan (2023) have applied this concept to consciousness, proposing that neural activity on the 4-dimensional “boundary” (the physical brain) has a mathematically equivalent description as a 5-dimensional “bulk” field—what Le Bihan terms the “C-field.” According to this model, what we experience as consciousness corresponds to excitations in this 5-dimensional field that emerge from 4-dimensional neural activity.

This framework offers, according to us, a new way to conceptualize Freud's topographic model. The unconscious could be understood not as physically “beneath” consciousness but as information encoded in the brain that lacks a corresponding excitation in the C-field. Repression of a mental representation might correspond to decoupling a bulk excitation (conscious awareness) from its boundary anchor (neural encoding), such that the information persists in the neural substrate but loses accessibility to consciousness.

While speculative, this approach aligns with Freud's intuition that unconscious content exists in a state that is neither physically separate from nor identical to conscious processes. The mathematics of the AdS/CFT correspondence potentially offers predictive metrics (such as bulk-to-boundary propagator norms) that could, theoretically, be mapped onto connectomic measurements of accessibility in memory recall experiments.

### 6.2 Dreaming and relativistic time

Regarding dreams, for quantum physics, Hobson et al. (2014) have proposed that dreaming represents a form of “virtual reality” in which the brain generates predictive models unconstrained by sensory input. This free-energy approach to dreaming aligns with the Feynman path integral analogy. The Feynman path integral is a way to understand how quantum particles move from point A to point B. Unlike a classical object that takes just one path, Feynman's approach says a quantum particle takes all possible paths simultaneously, each with its own probability. The final behavior of the particle emerges from adding up all these paths. Similarly, without the constraints of external reality-testing, the dreaming brain explores a wider probability space of mental configurations, much as path integrals sum over all possible quantum trajectories. In this analogy, waking consciousness can be compared to measurement in quantum mechanics, where observation selects one of many possible paths, transforming multiple quantum possibilities into a single observable reality.

This perspective enriches the Freudian conception where the manifest content of dreams represents an “observation” or reduction of a vast space of unconscious possibilities (latent content). The Freudian primary process, dominant in dreams (Freud, 2016), allows this superposition of contradictory and non-linear mental states, while waking and conscious interpretation act as a quantum measurement, selecting, and concretizing a specific psychic reality among all the potentialities that the unconscious had explored. Le Bihan's theory (2023; 2024) extends to dream states as well. Dreams could be understood as a kind of consciousness blocked in its interaction with the environment. It suggests that objective neural correlates might exist for subjective processes. This aligns with the search for Neural Correlates of Consciousness proposed by Crick (co-discoverer of the DNA structure) Crick and Koch (2003).

Another point of interest which this time concerns relativity is that Freud's work on dreams emphasized their non-logical, associative quality and their temporal fluidity (Freud, 2016). Einstein's special relativity established that time is not absolute but relative to the observer's reference frame. In dreams, subjective time often becomes elastic—moments can stretch to seem like hours, or hours can collapse into moments. Einstein himself wrestled with questions related to consciousness. In his 1921 Princeton lectures, Einstein (1956) noted: “The experiences of an individual appear to us arranged in a series of events; in this series the single events which we remember appear to be ordered according to the criterion of ‘earlier' and ‘later,' which cannot be analyzed further. There exists, therefore, for the individual, an 1-time, or subjective time. This in itself is not measurable.” Einstein thus recognized the gap between physical time and subjective time, gap particularly obvious in the case of dreams.

### 6.3 Observer participation and the analytic field

A fundamental insight of quantum mechanics is that observation is not passive but participatory. The observer and the observed form an inseparable system, and the act of measurement influences the phenomenon being measured. This observer effect fundamentally challenges the classical notion of objective, detached observation. Similarly, psychoanalysis recognizes that the analytic situation is not one where an objective analyst merely observes a patient's psyche. Rather, analyst and analysand form a co-constructed field, within which transference and countertransference create a unique intersubjective space. This parallel was first explored in the dialogue between physicist Wolfgang Pauli, Nobel price, and psychologist Carl Jung who was once close to Freud (Traversi and Mercier, 2018).

Their correspondence highlighted the symmetry between Bohr's complementarity principle in physics—which states that quantum entities can exhibit mutually exclusive properties depending on how they are measured—and the conscious-unconscious duality in analytical psychology (Traversi and Mercier, 2018).

In the analytic setting, an interpretation offered by the analyst can be understood as analogous to choosing a measurement basis in quantum physics. It actualizes one relational possibility at the expense of others, potentially altering both the analyst's and the analysand's mental states. Just as quantum measurement “collapses” multiple potential states into a single actualized state, an interpretation brings specific unconscious content into conscious awareness, reconfiguring the dynamics of the analytic field. Both fields support the same postulate: observation itself is a creative act that shapes reality (Vidal, 2014).

### 6.4 Between metaphor and model: the status of physical-psychoanalytic convergences

Having explored these convergences between Freudian concepts and contemporary physics, it is important to consider their epistemological status. Do these parallels represent mere metaphorical similarities, or do they point to deeper structural homologies? The current state of science does not yet allow us to definitively answer this question.

On one hand, the connections drawn here could be viewed as metaphorical extensions that help us conceptualize complex psychological phenomena using the language and formalism of physics. Metaphors can be powerful cognitive tools that generate new insights without necessarily implying identity between the domains being connected. From this perspective, concepts like the AdS/CFT correspondence or quantum measurement provide useful analogies for thinking about unconscious processes or the therapeutic relationship, without implying that psychic phenomena actually follow the same mathematical laws as physical systems.

On the other hand, these convergences might reflect deeper structural homologies. Both psychoanalysis and modern physics emerged during a period of profound epistemological shift at the turn of the twentieth century. Both disciplines challenged prevailing assumptions about the nature of reality, suggesting that there is more to the universe—whether physical or mental—than what directly appears to consciousness. This shared epistemological gesture may reflect common structural patterns that manifest across different scales and domains of reality.

Vidal (2014) argued that Freud's work—like that of contemporary physicists such as Einstein, Planck, and Bohr—participated in a fundamental ontological rupture with classical thought. Both psychoanalysis and modern physics moved away from naive realism toward a recognition that reality is constructed through complex processes that are not immediately accessible to conscious perception.

Regardless of whether these parallels represent metaphors or deeper homologies, their heuristic fertility suggests they merit serious consideration. The ability to translate between psychological and physical frameworks may generate novel hypotheses and research directions that would not emerge from either discipline in isolation.

It may be premature or vain to claim that contemporary physics “validates” Freudian theory in a direct sense. However, the resonances between these domains suggest that Freud's intuitions about psychic structure and function may have captured something fundamental about how complex information-processing systems—whether minds or quantum fields—organize and transform information across different levels of accessibility.

## 7 Conclusion

This interdisciplinary exploration of Freudian concepts through the lens of contemporary physics and neuroscience reveals that scientific advances offer new frameworks for understanding the phenomena that Freud described in psychological terms. Modern imaging technologies, particularly diffusion MRI, reveal the neurobiological foundations of phenomena that Freud intuitively addressed, while the cerebral plasticity observable in response to psychotherapeutic interventions challenges the traditional separation between organic and functional disorders.

The neurobiological mechanisms underlying memory suppression provide concrete correlates to the psychoanalytic conception of repression. Studies of conversion disorder confirm Freud's insight about the body's capacity to express psychological conflict through functional symptoms (Feinstein and Voon, 2021), and research on hypnosis reveals the neural basis for altered states of consciousness that he clinically explored (De Pascalis, 2024).

However, it is important to acknowledge several limitations to this integrative approach. First, the use of quantum physics and concepts such as the AdS/CFT duality (Awret, 2022) to illuminate psychic processes risks remaining at a metaphorical level rather than establishing verifiable structural homologies. The parallels between observation in quantum mechanics and the analytic situation, or between time relativity and dream experience, may constitute fruitful heuristic analogies without demonstrating true epistemological convergence.

Second, the cognitive unconscious studied by neuroscience fundamentally differs from the dynamic, affectively charged unconscious of psychoanalysis (Appourchaux, 2014). This conceptual difference raises the question of the commensurability of explanatory frameworks. Third, any attempt to explain complex psychological phenomena through neurobiological mechanisms or physical principles risks succumbing to reductionism, neglecting the phenomenological richness of subjective experience.

Our approach does not aim to reduce psychoanalysis to neurobiology or physics, but rather proposes an interdisciplinary dialogue where each field maintains its specificity while mutually enriching one another. What emerges is a more nuanced understanding of mental life that recognizes both its neurobiological foundations and its emergent psychological properties. As Naccache observed, “Freud's consciousness did not discover the unconscious; it invented it” (Naccache, 2022). This invention nevertheless opened pathways of investigation that continue to bear fruit when placed in dialogue with contemporary science.

Future research might further explore the relationship between psychoanalytic treatment and alterations in the connectome, developing more sophisticated models for understanding how specific psychoanalytic interventions modify brain structure and function. The intersection of quantum field theory and consciousness studies also remains promising, particularly as researchers develop more precise ways to test theories like Le Bihan's 5D model (Le Bihan, 2023) in relation to unconscious processes.

We are perhaps at the dawn of a new understanding that Freud, with his commitment to rigorous observation and theoretical innovation, would recognize and appreciate—an understanding that does not seek to definitively resolve the division between materialism and dualism, but rather to explore the multiple levels of organization that characterize the human mind.
